# A Rapid Spheroidizing Annealing Process for High-Carbon Steel

**DOI:** 10.3390/ma19020249

**Published:** 2026-01-08

**Authors:** Bei Li, Zhi Tong, Mengying Zhao, Xinlang Wu, Wenyue Zheng

**Affiliations:** 1 Research Institute of Technology, Shougang Group Co., Ltd., Beijing 100041, China; 2 National Center for Materials Service Safety, University of Science and Technology, Beijing 100083, China; 3 Beijing Shougang Co., Ltd., Beijing 100043, China

**Keywords:** high carbon steel, spheroidizing annealing, pre-treatment, hardness

## Abstract

Spheroidizing annealing is a critical heat treatment process for high-carbon steels to balance hardness and machinability. This study develops a rapid spheroidizing annealing process by employing low-temperature pretreatment followed by subcritical heating. The key is to utilize carbide precipitates from non-equilibrium phases (e.g., martensite/lower bainite) as nucleation sites, thereby accelerating spheroidization. At an optimized pretreatment temperature of 400 °C, the process achieves a homogeneous spheroidized microstructure with a hardness of 206.7 HV, comparable to that obtained via conventional prolonged annealing. This method significantly reduces processing time and energy consumption.

## 1. Introduction

High-carbon steels (typically containing 0.6–1.4 wt.% C) are widely employed in cutting tools, molds, bearings, and high-strength components due to their high hardness and wear resistance [1,2,3,4,5]. However, their high carbon content also leads to inherent brittleness and poor cold workability [1]. To balance the conflict between hardness and machinability, spheroidizing annealing serves as a critical step in the heat treatment process chain for high-carbon steels. This process involves precise control of the heating temperature, the holding time, and the cooling rate to promote the decomposition and reconstruction of lamellar pearlite or networked cementite in the original microstructure. By spheroidizing annealing lamellar pearlite transforms into fine spherical or granular carbide particles that are uniformly dispersed and embedded within the ferrite matrix [6]. In industrial manufacturing, one of the typical spheroidizing annealing processes is subcritical spheroidizing annealing [7]. This technique involves heating the steel slightly below the A1 temperature, holding it within the α + θ two-phase resgion (where only ferrite (α) and cementite (θ) are stable) for several hours, followed by cooling to room temperature. Throughout this entire spheroidizing annealing process, no austenite formation occurs. In contrast, another spheroidizing method known as intercritical spheroidizing annealing is employed. This process involves heating steel to a temperature slightly above A1 for austenitizing while retaining a small amount of undissolved cementite. After being held at this temperature for several hours, the steel is cooled to the α + θ phase region (temperature just below A1). Compared to the pearlite reaction, this process of directly producing spherical carbides from austenite (γ) is popularly known as the divorced eutectoid transformation (DET) reaction [7].

In the case of ultra-high carbon steel (UHCS), the spheroidization process based on the divorced eutectoid transformation mechanism has been given significant attention in recent years [8,9,10,11,12]. Researches [13,14] indicate that the initial microstructure plays a decisive role in the spheroidization process. For instance, degenerated pearlite (D-P) [1], characterized by its finer interlamellar spacing, demonstrates an accelerated carbide dissolution rate during spheroidizing annealing, thereby enhancing spheroidization kinetics. As demonstrated by Li et al. [15], precise regulation of the cooling rate within a defined window between the austenitizing and secondary annealing processes is essential to suppress the precipitation of lamellar carbides (LC). Through optimization of these interlinked thermal parameters, a homogeneous carbide distribution accompanied by reduced material hardness can be effectively achieved.

Meanwhile, research on spheroidizing annealing has progressively advanced toward more efficient and energy-saving methodologies. Han et al. [16] confirmed that the precipitation of deformation-induced carbides in bearing steel effectively reduces the carbon and chromium content in the austenitic matrix. This compositional modification alters both the phase transformation driving force and interfacial energy, thereby creating favorable thermodynamic conditions for the carbide spheroidization process. Qian et al. [17] conducted in-depth research on 52100 bearing steel, revealing the synergistic mechanism between divorced eutectoid transformation and plastic deformation in promoting microstructural spheroidization. Under suitable warm working conditions, plastic strain increases dislocation density, providing additional nucleation sites for carbide precipitation, while the divorced eutectoid transformation promotes the formation of spherical carbides by modifying phase boundary characteristics. Besides, investigation into the application of air quenching as a pretreatment technique demonstrates its effectiveness in fragmenting coarse plate-like carbides in the initial microstructure [18]. This preliminary microstructural optimization enhances interfacial area and strain energy, providing stronger kinetic driving forces for subsequent spheroidization. This approach not only significantly accelerates the spheroidization process but also yields a more homogeneous and stable final microstructure.

Although the divorced eutectoid transformation (DET) provides an efficient spheroidization pathway, it typically requires high-temperature pretreatment to generate undissolved carbides as nucleation sites. To explore alternative nucleation pathways and further enhance process efficiency, this study proposes a novel strategy employing low-temperature pretreatment to obtain non-equilibrium phases (martensite/lower bainite), followed by continuous heating to a subcritical temperature. This approach aims to utilize the fine carbides precipitated during the decomposition of these metastable phases as potent nucleation sites, thereby achieving rapid spheroidization while minimizing energy consumption and total process time.

## 2. Materials and Methods

**Figure 1:**
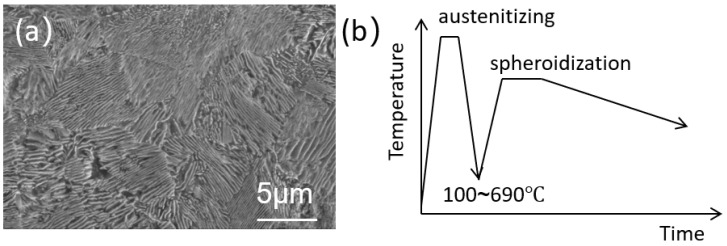
(**a**) Microstructure of ultra-high-strength pipeline steel; (**b**) Thermo-mechanical control process schedule.

In this work, the experimental steel was taken from hot-rolled steel plates with the chemical composition shown in Table 1. And the experimental steel was prepared by thermo-mechanical control process (TMCP). As shown in Figure 1a, the microstructure of high-carbon steel was mainly fine lamellar pearlites, and the hardness value is approximately 320 HV. The thermomechanical control process schedule is displayed in Figure 1b.

**Figure 2:**
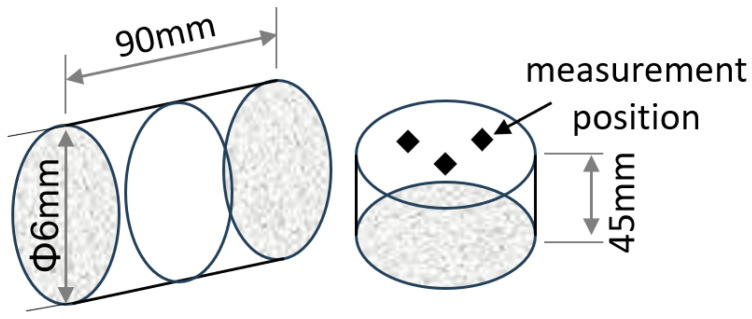
Sampling locations and SEM measurement positions.

The experiment employed the following thermal cycle: ① Heating to 880 °C at 10 °C/s and holding for 3 min to achieve complete austenitizing; ② Cooling to 100 °C, 300 °C, 400 °C, 500 °C, 550 °C, 600 °C, 650 °C, and 700 °C, respectively, at 10 °C/s to obtain pretreated microstructures with varying characteristics; ③ Reheating to 710 °C at 10 °C/s, followed by slow cooling to 200 °C at 0.05 °C/s. This cycle simulated a high-temperature reheating and slow cooling process to achieve optimized spheroidizing annealing results. The experiments were conducted using a Gleeble—3800 thermal simulation system (Dynamic Systems Inc., Poestenkill, NY, USA) and the quenching medium is compressed air. Microstructural characterization and analysis were performed by scanning electron microscopy (SEM, Thermoscientific-Apro 2S, Waltham, MA, USA), with the specific specimen and measurement locations shown in Figure 2. Vickers hardness was measured under an HV10 load in accordance with the GB/T 4340.1 standard [19].

## 3. Results and Discussion

### 3.1. Microstructures

**Figure 3:**
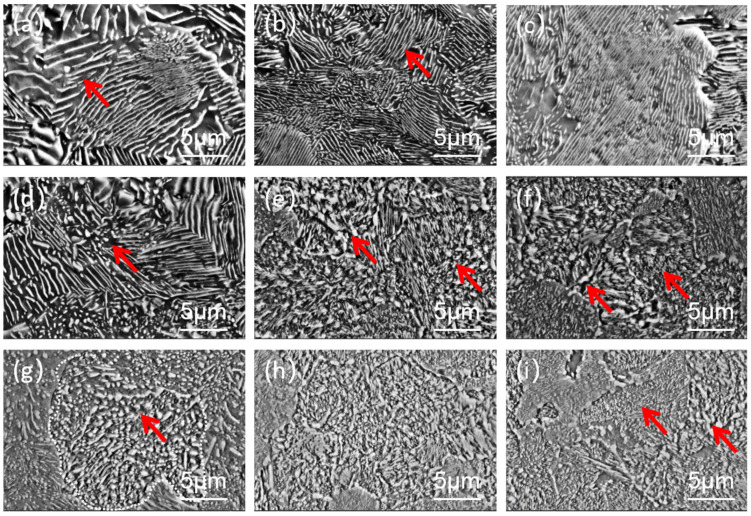
Microstructures after rapid spheroidizing annealing: Pretreatment temperatures from (**a**–**f**) are 690 °C, 660 °C, 630 °C, 600 °C, 550 °C, and 500 °C, respectively, while (**g**–**i**) correspond to 400 °C, 300 °C, and 100 °C. The red arrow represents a typical pearlite.

Figure 3 displays the microstructures after heat treatment. As shown in Figure 3a–c, the pretreatment temperatures ranged from 630 °C to 690 °C. Within this temperature range, the microstructure remains lamellar pearlite. This is because the high pretreatment temperature has already promoted the formation of lamellar pearlite (with an interlamellar spacing of 207~1350 nm). And the interlamellar spacing at different temperatures is shown in Table 2. Additionally, the short holding time during the subsequent reheating stage is insufficient to allow the spheroidization of the pearlite.

Figure 3d–f reveals that, within the pretreatment temperature range of 500–600 °C, the microstructure consists of a mixed structure comprising of lamellar pearlites and spheroidized pearlites. At 600 °C, a high proportion of distinct lamellar pearlites and a small amount of spheroidized pearlites are observed. When the temperature decreases to 550 °C, the proportion of spheroidized pearlites increases, exhibiting large-sized granular characteristics. This is primarily attributed to the inheritance effect during subsequent reheating, resulting from the initial coexistence of upper bainite and lamellar pearlite. As the temperature further drops to 500 °C, only trace amounts of lamellar pearlites and spheroidized pearlites with a multi-scale size distribution remain. The formation mechanism can be ascribed to: the continued coarsening of upper bainite granular structures formed during pretreatment, concurrent with the continuous precipitation and growth of carbides in lower bainite. The heterogeneity in the size of the primary carbides ultimately leads to the formation of a multi-scale mixed structure.

As shown in Figure 3g, when the pretreatment temperature is 400 °C, the microstructure predominantly consists of fine granular carbides uniformly distributed in the ferrite matrix. This morphological characteristic is presumably attributed to the lower bainite structure obtained during pretreatment: during the subsequent reheating process, α-Fe precipitates fine-scale carbides, which subsequently undergo direct growth and coarsening with these precipitates as nucleation sites.

When the pretreatment temperature is further reduced to 100–300 °C (Figure 3h,i), the microstructure exhibits spheroidized pearlite with extremely fine particle sizes. This phenomenon can be reasonably attributed to the following mechanism: the martensitic structure obtained from pretreatment undergoes carbide precipitation during reheating, and these precipitates subsequently act as nucleation sites for further growth.

Comparative analysis of the experimental processes reveals that when lamellar pearlite forms during the pretreatment stage, subsequent reheating struggles to achieve complete spheroidization within a short duration. This is primarily due to the high thermal stability of the lamellar cementite (Fe_3_C). In contrast, when the pretreatment temperature is controlled within a lower range, the reheating process promotes uniform nucleation and precipitation of carbides, facilitating the formation of spherical or near-spherical pearlite. However, when the pretreatment temperature is too low, the carbides precipitated from martensite decomposition are excessively fine, requiring more energy to growth. This consequently leads to a significant extension of the process duration needed to obtain spherical carbides of suitable size.

### 3.2. Hardness Tests

**Figure 4:**
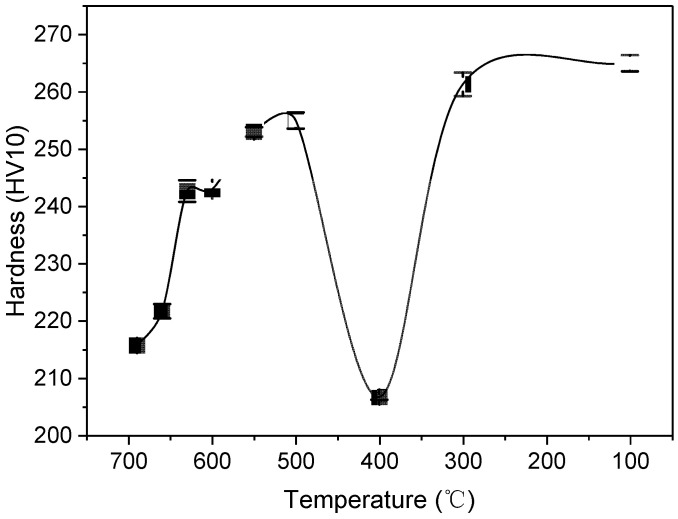
Variation of hardness with the pretreatment temperature.

The Vickers hardness of the annealed samples shows a non-monotonic dependence on the pretreatment temperature (Figure 4). As the temperature decreases from 690 °C to 500 °C, the hardness increases progressively from 215.7 HV to 255 HV. Notably, a further reduction to 400 °C leads to a pronounced hardness drop to 206.7 HV. However, a further decrease to 400 °C results in an unexpected and significant drop in hardness to 206.7 HV. As the temperature continues to decrease to 200 °C, the hardness rises again to 261.3 HV. At a pretreatment temperature of 100 °C, the hardness reaches a peak value of 265 HV, showing no significant difference compared to the result at 200 °C.

These results clearly reveal two distinct hardness troughs at specific pretreatment temperatures: 690 °C and 400 °C. Microstructural analysis provides a reasonable explanation for this phenomenon. The final microstructure obtained at 690 °C consists of coarse pearlite with a large interlamellar spacing, a typical coarse structure that generally leads to lower strength and hardness properties. At 400 °C, the microstructure comprises uniformly sized spheroidized pearlite of approximately 400 nm. Although spheroidized pearlite often offers a good combination of properties, it demonstrated relatively low hardness characteristics at this specific size and morphology, leading to the second observed hardness trough.

### 3.3. Spheroidization Mechanism

**Figure 5:**
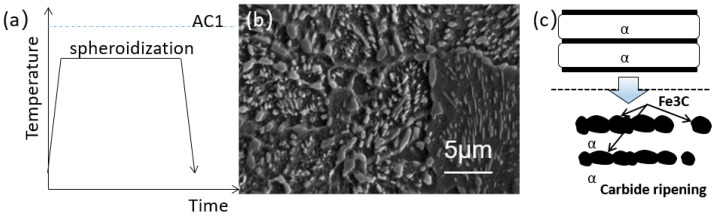
(**a**) Flowchart of conventional spheroidizing annealing; (**b**) Microstructure after holding at 710 °C for 14 h; (**c**) Mechanism of conventional spheroidizing annealing.

For samples subjected to higher pretreatment temperatures, where lamellar pearlite has already formed, prolonged holding time is typically required to achieve complete spheroidization. As shown in Figure 5a, maintaining the temperature at 710 °C for 14 h is necessary to achieve a hardness of 198 HV (range: 182–204 HV). The corresponding microstructure is presented in Figure 5b.

This phenomenon can be attributed to the complex morphological evolution of carbides during spheroidizing annealing, which involves both thermodynamic driving forces and kinetic controls. The core of this process lies in phase transformation regulation to achieve the transition from plate-like cementite to spherical or granular carbides. The process follows primarily the principle of interfacial energy minimization [2,20,21]. Thermodynamically, non-spherical lamellar carbides possess higher specific surface area and interfacial energy. Under prolonged high-temperature holding, the system tends to reduce the total interfacial energy to reach a more stable state, thereby promoting the spheroidization of carbides.

This evolution generally occurs in three stages, as illustrated in Figure 5c: Firstly, the fragmentation and breakage of cementite within the lamellar pearlite; Secondly, the spheroidization and coarsening (Ostwald ripening) of carbide particles; Finally, the formation of uniformly dispersed spherical carbides. Studies have indicated that degenerated pearlite (D-P) [1] with finer interlamellar spacing exhibits significantly accelerated carbide dissolution and spheroidization rates during subcritical spheroidizing annealing. This is attributed to the shorter diffusion path for carbon atoms provided by the reduced interlamellar spacing, which enhances the instability of the Fe_3_C phase and accelerates its fragmentation and evolution into spherical particles. However, once lamellar pearlites are formed, an extended duration remains essential for its transformation into spheroidized/granular pearlite.

Based on the mechanism of Divorced Eutectoid Transformation (DET)-driven spheroidization annealing [22], this process involves two key stages: first, austenitizing the steel within the three-phase (γ + α + carbide) region to produce a partially transformed microstructure containing undissolved cementite particles; second, holding the material at a sub-eutectoid temperature to promote carbide spheroidization. The undissolved cementite present after intercritical annealing significantly accelerates subsequent spheroidization by facilitating rapid carbon dissolution and re-precipitation. This mechanism explains the high efficiency of the DET-based process in achieving a uniform dispersion of ultrafine spheroidized carbides, making it particularly suitable for industrial spheroidizing annealing applications where processing time and microstructural homogeneity are critical. In the process of achieving rapid spheroidization annealing, lamellar pearlite should be strictly avoided, as its formation significantly prolongs the duration required for pearlite spheroidization, as illustrated in Figure 5. Addressing this issue, Li et al. [15] specifically demonstrated that controlling the cooling rate between austenitizing and secondary annealing within a specific range is crucial to prevent the formation of lamellar carbide (LC). Their research confirms that an appropriate cooling rate promotes more uniform carbide distribution, reduces dislocation density, and results in lower material hardness.

**Figure 6:**
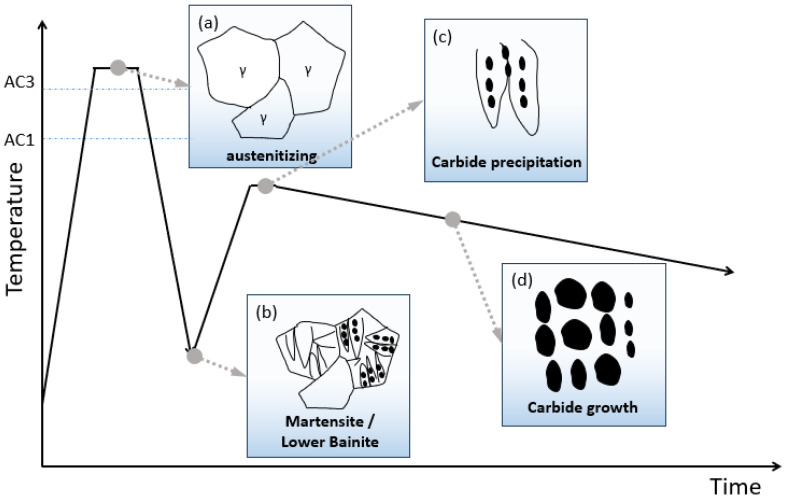
Schematic illustration of spheroidization mechanism: (**a**) Heated to the full austenitizing state; (**b**) Rapidly cooled to the low-temperature transformation zone to obtain a microstructure comprising martensite/lower bainite; (**c**) Decomposition of metastable phases yields carbide precipitates that serve as nucleation sites for spheroidized pearlite; (**d**) The spheroidized pearlite structure is obtained through carbide growth.

Building upon rigorous theoretical re-examination of the divorced eutectoid transformation (DET) framework, we propose a refined mechanistic model for spheroidized pearlite formation, fundamentally conceptualized as a two-stage process comprising nucleation and subsequent growth. Within this paradigm, undissolved carbides serve as preferential nucleation sites—a premise that prompted critical theoretical inquiry into alternative pathways for carbide precursor engineering. This theoretical foundation motivated our experimental design, specifically targeting low-temperature metastable phase structures as precursor matrices. The underlying hypothesis posits that the controlled precipitation within these metastable phases can be strategically harnessed to generate spheroidization nuclei with enhanced catalytic efficiency. From a microstructure selection perspective, martensitic and lower bainitic structures were identified as optimal candidates due to their characteristic non-equilibrium phase configurations and inherent driving forces for transformation. To quantitatively delineate the microstructural evolution pathways, we present a comprehensive schematic (Figure 6) that contrasts our proposed mechanism against both conventional spheroidization annealing and established DET-based processes, highlighting fundamental differences in nucleation kinetics and growth thermodynamics. The theoretical framework further establishes that the nucleation barrier for spheroidized pearlite is substantially reduced when catalyzed by pre-existing precipitation within metastable phases, while the growth kinetics follow a diffusion-controlled regime governed by carbon partitioning between the carbide interfaces and ferritic matrix.

By strategically leveraging the controlled decomposition of intentionally induced non-equilibrium phases—specifically martensite and lower bainite—an innovative and highly efficient spheroidization pathway has been successfully established, achieving remarkable microstructural refinement within significantly reduced processing time. This advanced methodology operates through two fundamentally interconnected and mutually reinforcing mechanisms that collectively drive the accelerated spheroidization kinetics. Primarily, the nanometer-scale transition carbides precipitated during the initial stages of martensite/lower bainite decomposition—particularly carbides in martensite and fine cementite in bainite—serve as ideal nucleation substrates for subsequent spheroidized pearlite formation, effectively bypassing the stochastic nucleation barrier encountered in conventional approaches. Secondly, and perhaps more critically, the substantial crystal defects generated during phase decomposition—including the excess vacancies and high-density dislocation networks released during the decomposition process enhance carbon mobility [23,24,25]. This unique microstructural environment not only facilitates rapid carbon supersaturation adjustment but also enables directional dissolution and redistribution of carbides through accelerated pipe diffusion along dislocations, ultimately resulting in spheroidized microstructures with refined carbide dispersion and optimal interfacial characteristics.

## 4. Conclusions

This study established an efficient spheroidizing annealing route for high-carbon steel by controlling the pretreatment temperature, leading to the following main conclusions:

(1) Controlling the pretreatment temperature within a lower range (e.g., 400 °C) promotes uniform precipitation of fine carbides during subsequent reheating, which facilitates the formation of a spherical or near-spherical pearlite microstructure. When the pretreatment temperature is too low, the carbides precipitated from martensite decomposition are excessively fine, requiring additional energy to drive their Ostwald ripening process. This consequently leads to a significant extension of the process duration needed to obtain spherical carbides of suitable size.
(2) As the pretreatment temperature decreases from 690 °C to 100 °C, the hardness exhibits an initial increase, followed by a decrease at 400 °C, and then a subsequent increase. The significant hardness reduction to 206.7 HV at 400 °C represents an ideal hardness level comparable to that achieved through conventional spheroidizing annealing.
(3) The decomposition of induced non-equilibrium phases (martensite/lower bainite) establishes an efficient spheroidization pathway. The fine carbides precipitated during phase transformation provide nucleation sites for spheroidized pearlite growth, while the excess vacancies and high-density dislocation networks released during decomposition enhance carbon mobility, ultimately promoting the spheroidization process. This process achieves significant microstructure refinement while significantly shortening the processing time. It will help save energy and time in industrial production.

## Figures and Tables

**Table 1:** Chemical composition of experimental steel (wt.%).

C	Si	Mn	Cr	Fe
≤0.80	≤0.3	≤0.80	≤0.20	-

**Table 2:** Pearlite Interlamellar Spacing Measurement (nm).

Temperature/°C	690	660	630
Interlamellar Spacing/nm	480~1350	304~660	207~500

## Data Availability

The original contributions presented in this study are included in the article. Further inquiries can be directed to the corresponding authors.
